# Fauna Europaea: Diptera – Brachycera

**DOI:** 10.3897/BDJ.3.e4187

**Published:** 2015-02-20

**Authors:** Thomas Pape, Paul Beuk, Adrian Charles Pont, Anatole I. Shatalkin, Andrey L. Ozerov, Andrzej J. Woźnica, Bernhard Merz, Cezary Bystrowski, Chris Raper, Christer Bergström, Christian Kehlmaier, David K. Clements, David Greathead, Elena Petrovna Kameneva, Emilia Nartshuk, Frederik T. Petersen, Gisela Weber, Gerhard Bächli, Fritz Geller-Grimm, Guy Van de Weyer, Hans-Peter Tschorsnig, Herman de Jong, Jan-Willem van Zuijlen, Jaromír Vaňhara, Jindřich Roháček, Joachim Ziegler, József Majer, Karel Hůrka, Kevin Holston, Knut Rognes, Lita Greve-Jensen, Lorenzo Munari, Marc de Meyer, Marc Pollet, Martin C. D. Speight, Martin John Ebejer, Michel Martinez, Miguel Carles-Tolrá, Mihály Földvári, Milan Chvála, Miroslav Barták, Neal L. Evenhuis, Peter J. Chandler, Pierfilippo Cerretti, Rudolf Meier, Rudolf Rozkosny, Sabine Prescher, Stephen D. Gaimari, Tadeusz Zatwarnicki, Theo Zeegers, Torsten Dikow, Valery A. Korneyev, Vera Andreevna Richter, Verner Michelsen, Vitali N. Tanasijtshuk, Wayne N. Mathis, Zdravko Hubenov, Yde de Jong

**Affiliations:** ‡ Natural History Museum of Denmark, Copenhagen, Denmark; § Natural History Museum Maastricht / Diptera.info, Maastricht, Netherlands; | Oxford University Museum of Natural History, Oxford, United Kingdom; ¶ Zoological Museum, Moscow State University, Moscow, Russia; # Wrocław University of Environmental and Life Sciences, Wrocław, Poland; ¤ Muséum d'histoire naturelle Genève, Paris, Switzerland; « Forest Research Institute, Department of Forest Protection, Warszawa, Poland; » Tachinidae Recording Scheme, London, United Kingdom; ˄ Unaffiliated, Uppsala, Sweden; ˅ Senckenberg Natural History Collections Dresden, Museum of Zoology, Dresden, Germany; ¦ Unaffiliated, Cardiff, United Kingdom; ˀ CABI, Sussex, United Kingdom; ˁ I.I. Schmalhausen Institute of Zoology, Kiev, Ukraine; ₵ Zoological Institute Russian Academy of Sciences, St Petersburg, Russia; ℓ Department of Forensic Medicine, University of Copenhagen, Copenhagen, Denmark; ₰ Unaffiliated, Braunschweig, Germany; ₱ Zoological Museum, Zürich, Switzerland; ₳ Museum Wiesbaden, Natural History Collections, Wiesbaden, Germany; ₴ Unaffiliated, Reet (Rumst), Belgium; ₣ Staatliches Museum für Naturkunde, Stuttgart, Germany; ₮ NBC Naturalis, Leiden, Netherlands; ₦ Unaffiliated, Waalwijk, Netherlands; ₭ Masaryk University, Brno, Czech Republic; ₲ Silesian Museum, Opava, Czech Republic; ‽ Museum für Naturkunde, Berlin, Germany; ₩ University of Pécs, Pécs, Hungary; ₸ Charles University, Prague, Czech Republic; ‡‡ Natural History Museum of Sweden, Stockholm, Sweden; §§ University of Stavanger, Stavanger, Norway; || Zoological Museum, Oslo, Norway; ¶¶ c/o Natural History Museum, Venice, Italy; ## Royal Museum for Central Africa, Tervuren, Belgium; ¤¤ Research Institute for Nature and Forest, Brussels, Belgium; «« Dept. of Zoology, Trinity College, Dublin 2, Ireland; »» Unaffiliated, Cowbridge, United Kingdom; ˄˄ INRA, UMR Centre de Biologie pour la Gestion des Populations, Montferrier-sur-Lez, France; ˅˅ Unaffiliated, Barcelona, Spain; ¦¦ MTA-DE ‘Lendület’ Behavioural Ecology Research Group, Department of Evolutionary Zoology, University of Debrecen, Debrecen, Hungary; ˀˀ Czech University of Life Sciences, Prague, Czech Republic; ˁˁ Bishop Museum, Honolulu, United States of America; ₵₵ Unaffiliated, Melksham, United Kingdom; ℓℓ University of Rome, Roma, Italy; ₰₰ National University of Singapore, Singapore, Singapore; ₱₱ California Department of Food and Agriculture, Sacramento, California, United States of America; ₳₳ Department of Biosystematics, Opole University, Opole, Poland; ₴₴ Unaffiliated, Soest, Netherlands; ₣₣ National Museum of Natural History, Smithsonian Institution, Washington DC, United States of America; ₮₮ National Museum of Natural History, Sofia, Bulgaria; ₦₦ University of Eastern Finland, Joensuu, Finland; ₭₭ University of Amsterdam - Faculty of Science, Amsterdam, Netherlands

**Keywords:** Biodiversity Informatics, Fauna Europaea, Taxonomic indexing, zoology, biodiversity, taxonomy, Diptera, Brachycera

## Abstract

*Fauna Europaea* provides a public web-service with an index of scientific names (including important synonyms) of all extant multicellular European terrestrial and freshwater animals and their geographical distribution at the level of countries and major islands (east of the Urals and excluding the Caucasus region). The *Fauna Europaea* project comprises about 230,000 taxonomic names, including 130,000 accepted species and 14,000 accepted subspecies, which is much more than the originally projected number of 100,000 species. *Fauna Europaea* represents a huge effort by more than 400 contributing taxonomic specialists throughout Europe and is a unique (standard) reference suitable for many user communities in science, government, industry, nature conservation and education. The Diptera–Brachycera is one of the 58 *Fauna Europaea* major taxonomic groups, and data have been compiled by a network of 55 specialists.

Within the two-winged insects (Diptera), the Brachycera constitute a monophyletic group, which is generally given rank of suborder. The Brachycera may be classified into the probably paraphyletic 'lower brachyceran grade' and the monophyletic Eremoneura. The latter contains the Empidoidea, the Apystomyioidea with a single Nearctic species, and the Cyclorrhapha, which in turn is divided into the paraphyletic 'aschizan grade' and the monophyletic Schizophora. The latter is traditionally divided into the paraphyletic 'acalyptrate grade' and the monophyletic Calyptratae. Our knowledge of the European fauna of Diptera–Brachycera varies tremendously among families, from the reasonably well known hoverflies (Syrphidae) to the extremely poorly known scuttle flies (Phoridae). There has been a steady growth in our knowledge of European Diptera for the last two centuries, with no apparent slow down, but there is a shift towards a larger fraction of the new species being found among the families of the nematoceran grade (lower Diptera), which due to a larger number of small-sized species may be considered as taxonomically more challenging.

Most of Europe is highly industrialised and has a high human population density, and the more fertile habitats are extensively cultivated. This has undoubtedly increased the extinction risk for numerous species of brachyceran flies, yet with the recent re-discovery of *Thyreophora cynophila* (Panzer), there are no known cases of extinction at a European level. However, few national Red Lists have extensive information on Diptera.

For the Diptera–Brachycera, data from 96 families containing 11,751 species are included in this paper.

## Introduction

In 1998 the European Commission published the European Community Biodiversity Strategy, providing a framework for the development of Community policies and instruments in order to comply with the Convention on Biological Diversity. The Strategy recognised the current incomplete state of knowledge at all levels of biodiversity, a state which makes a successful implementation of the Convention difficult. *Fauna Europaea* was conceived to contribute to this Strategy by supporting one of the main themes: to identify and catalogue the components of the European biodiversity, with the cataloguing implemented as a taxonomic and faunistic database serving as a basic tool for scientific documentation and discovery, environmental management, and conservation policies/priorities.

With regard to biodiversity in Europe, science and policies depend on sufficient knowledge of the relevant components. The assessment of biodiversity, including monitoring changes and ensuring sustainable exploitation, as well as much legislative work, depend upon a validated taxonomic overview, in which *Fauna Europaea* will play a major role by providing a web-based information infrastructure with an index of scientific names (including the most important synonyms) of all living European multicellular terrestrial and freshwater animals, their geographical distribution at the level of countries and major islands, and some relevant additional information.

*Fauna Europaea* (FAEU) kicked off in 2000 as an EC-FP5 four-year project, delivering its first release in 2004 (de Jong et al. 2014). This online-only version has continuously been updated, and after a further decade of steady progress, to efficiently disseminate the results of *Fauna Europaea* and to properly credit the *Fauna Europaea* contributors, modern e-publishing tools are being applied to prepare data papers on all 58 major taxonomic groups. For this purpose a special Biodiversity Data Journal Series has been compiled, called Contributions on Fauna Europaea (see also: Pensoft News item 17 Dec 2014). This work was initiated during the ViBRANT project and is further supported by the recently started EU BON project. This paper is the first publication from the *Fauna Europaea* Diptera–Brachycera data sector as a BDJ data paper in the *Fauna Europaea* series, and further contributions should be expected when warranted by major updates.

In the EU BON project (Hoffmann et al. 2014) further steps will be made to implement *Fauna Europaea* as a basic tool and standard reference for biodiversity research and as a means to facilitate taxonomic expertise evaluation and management in Europe. The *Fauna Europaea* data papers will contribute to a quality assessment on biodiversity data by providing estimates on gaps in our taxonomic information and knowledge.

## General description

### Purpose

*Fauna Europaea* is a database of the scientific names and distributions (at national or in some cases regional level) of all currently known extant multicellular European terrestrial and freshwater animal species. The database has been assembled by a large network of taxonomic specialists. An extended description of the *Fauna Europaea* project can be found in de Jong et al. 2014. A summary is given in the sections below.

The Diptera–Brachycera is one of the 58 *Fauna Europaea* major taxonomic groups, covering 11,751 species (Table 1), and the data have been gathered by a network of 55 specialists (Tables 1, 3).

### Additional information

**Diptera**–**Brachycera**

Diptera are usually classified into the 'nematoceran grade' or 'lower Diptera' and the monophyletic Brachycera. The Brachycera may in turn be classified into the probably paraphyletic 'lower Brachycera' and the monophyletic Eremoneura. The latter contains the Empidoidea, the Apystomyioidea with a single Nearctic species, and the Cyclorrhapha, which in turn are divided into the paraphyletic 'aschizan grade' and the monophyletic Schizophora. The latter are traditionally divided into the paraphyletic 'acalyptrate grade' and the monophyletic Calyptratae (Yeates and Wiegmann 1999, Wiegmann et al. 2011, Lambkin et al. 2013). Diptera increase in the relative proportion of the insect fauna at increasing altitude as well as at higher latitudes, whether counting the number of species or the number of individuals. In Europe, Diptera are surpassed only by the Hymenoptera in the total number of species, but Diptera are the predominant insect group in high montane, subarctic, and arctic environments. Europe lies mainly in the temperate climate zone, and its species diversity is relatively poor, being heavily influenced by the Quaternary glaciations. With some 12,000 species of Brachycera and 7,000 species of the nematoceran grade (lower Diptera), the European fauna of Diptera is comparable to those of the Nearctic (ca. 22,000), Afrotropical (ca. 20,000), Oriental (ca. 23,000) and Australasian (ca. 19,000) regions (Pape et al. 2009). The knowledge of the taxonomic composition of the European Diptera fauna may therefore be considered as far more complete than for any other major region. This relates to historical circumstances, with Europe having a much longer taxonomic tradition and with relatively more funding being available to the European taxasphere. The number of species added to the European Diptera fauna has been remarkably constant over time, and the species accumulation curve shows to date no signs of levelling off (Pape 2009, Fontaine et al. 2012, Pape, unpubl.). Among the Brachycera, the most species-rich families in the European fauna are the Agromyzidae, Dolichopodidae, Empididae, Syrphidae and Tachinidae. Much remains to be discovered, and especially the Phoridae stand out as potentially vastly more diverse than suggested by the current count.

Brachycera are ecologically very diverse (Hövemeyer 2000). Many of the 'lower Brachycera' are predatory in the larval stage, with the parasitic Acroceridae, Bombyliidae and Nemestrinidae as significant exceptions. The Empidoidea include a large assemblage of species with predatory adult and larval stages. Many lineages within the species-rich Cyclorrhapha have adapted to a saprophagous larval life, but also parasitism and predation have evolved numerous times within this group, e.g., millipede parasitising Phaeomyiidae and Muscidae (in part: *Eginia* Robineau-Desvoidy); mollusc parasitising Sciomyzidae and Calliphoridae (in part, e.g., Melanomyinae); insect parasitising Cryptochetidae, Pipunculidae, Pyrgotidae, Tachinidae and Sarcophagidae (in part, e.g., *Blaesoxipha* Loew); woodlouse parasitising Rhinophoridae; mammal parasitising Oestridae; plant parasitising Agromyzidae, Tephritidae, Anthomyiidae (in part), Chloropidae (in part) and Scathophagidae (in part); and insect predating Chamaemyiidae, Chloropidae (in part), Muscidae (in part), Odiniidae (in part) and Syrphidae (in part). The genera *Nephrocerus* Zetterstedt (Pipunculidae) and *Admontia* Brauer & Bergenstamm and *Siphona* Meigen (both Tachinidae) deserve special mention because they contain species that are parasitoids of other Diptera (in this case Tipulidae), and *Nephrocerus* spp. may be the only European fly species that parasitise adult Diptera (Koenig and Young 2007). The Phoridae present a remarkable diversity of life habits, ranging from extreme specialisations like the ladybird parasitising species of *Phalacrotophora* Enderlein to the 'omnivorous' *Megaselia scalaris* (Loew), which has been bred from an astonishingly broad range of organic materials even including shoe polish and paint (Disney 1994). Shore flies (Ephydridae) are magnificently tolerant of extreme environments, such as hot springs, saline and alkaline waters, and even crude oil (references in Mathis and Zatwarnicki 1995). The Syrphidae are well known for the mimetism of many adults and the multitude of larval life forms, with some of the more classic examples including rat-tails living in putrid water, free-living aphid predators, bulb miners, and inquilines and scavengers in nests of ants, bees and social wasps (Thompson and Rotheray 1998).

Brachyceran flies contain several important agricultural pests, like cabbage flies (*Delia* spp., Anthomyiidae), shoot flies (*Atherigona* spp., Muscidae), frit flies [*Oscinella frit* (Linnaeus), Chloropidae], and fruit flies [e.g., *Ceratitis capitata* (Wiedemann) and *Bactrocera oleae* (Rossi), Tephritidae); others are blood-sucking, like the horn fly [*Haematobia irritans* (Linnaeus), Muscidae] and the false stable fly [*Muscina stabulans* (Fallén) Muscidae]; or vectors of various diseases like the bovine filariasis transmitted by some species of *Musca* Linnaeus (Krafsur and Moon 1997). Flies may be a nuisance when occurring in vast numbers around landfills, garbage dumps, or dung-heaps (Howard 2001). Particularly remarkable cases of mass occurrences are given by the chloropid fly *Thaumatomyia notata* (Meigen), specimens of which, possibly guided by a species-specific male pheromone, seek suitable places for overwintering and in extreme cases may enter buildings in such numbers that they darken the ceilings and create bucket-loads of dead bodies when they die in the dry indoor climate (Nartshuk 2000). On the beneficial side, many Brachycera are efficient decomposers and play an important role in cleaning sewage and recycling organic waste (McLean 2000); some hover flies (Syrphidae) and grass flies (Chloropidae) are predators on pest aphids (Ismay and Nartshuk 2000, Thompson and Rotheray 1998) and larvae of the long-legged fly genus *Medetera* (Dolichopodidae) feed on all stages of bark beetles (Curculionidae, Scolytinae) (Hulcr et al. 2005); and blow flies (Calliphoridae) may serve as forensic indicators (Catts and Goff 1992, Byrd and Castner 2000, Rivers and Dahlem 2014) and even improve human health through the treatment of complicated wounds (Sherman 2001, Sherman 2002, Sherman 2003). *Drosophila melanogaster* Meigen (Drosophilidae) has become the archetype of a geneticists laboratory animal, and the multitude of genetic studies performed on this species has had a profound impact on our understanding of gene expression, gene regulatory mechanisms, mutations, etc. (see references in Courtney et al. 2009).

One European brachyceran recently considered as extinct (Fontaine et al. 2007, Pape 2009) was rediscovered in Spain in this decade (Carles-Tolrá et al. 2010, Martín-Vega et al. 2010, Zaldivar Ezquerro et al. 2011, Carles-Tolrá and Cañete Saiz 2012): the bone-skipper *Thyreophora cynophila* (Panzer) (Piophilidae: Thyreophorinae) was, around 1800, frequently encountered on larger carrion like dogs, mules, and horses in very early spring (Séguy 1950). The present-day rareness of this morphologically quite conspicuous species may be due to changes in livestock management and improved carrion disposal following the Industrial Revolution in Europe. The growth in human population and the associated reduction in the number of large predators may also have played a part, as this has meant fewer large carcasses with partly crushed long bones, which appears to be one of the favoured breeding media for *T. cynophila*.

The distributional pattern of European Brachycera keeps changing, and the underlying causes may not always be evident. For example, the cold-adapted species *Scoliocentra nigrinervis* (Wahlgren) (Heleomyzidae) has been newly recorded from areas in Central Europe where it was previously unrecorded (Preisler and Roháček 2012, Soszyńska-Maj and Woźnica 2012). This is in contrast to the current climate change related to global warming during recent decades. Another species, *Prosopantrum flavifrons* (Enderlein) (Cnemospathididae) was probably accidentally introduced from South Africa by bird migrations to one of the British Isles (Ismay and Smith 1994, Cole 1996), and being a parthenogenetic species with larvae developing in bird guano, it has spread to one of the East Frisian Islands (Stuke and Merz 2005).

## Project description

### Title

This BDJ data paper includes the taxonomic indexing efforts in *Fauna Europaea* on European Diptera–Brachycera covering the first two versions of *Fauna Europaea* (up to version 2.6).

### Personnel

The taxonomic framework of *Fauna Europaea* includes scientists from the 34 partner institutes, which together with a number of citizen scientists provide the taxonomic expertise and faunistic quality assurance and take care of data collation.

Every taxonomic group is covered by at least one Group Coordinator responsible for the supervision and integrated input of taxonomic and occurrence data of a particular group. For Diptera–Brachycera the responsible Group Coordinators are Thomas Pape (versions 1 & 2) and Paul Beuk (version 2).

The *Fauna Europaea* checklist would not have reached its current level of completion without the input from several taxonomic specialists. The formal responsibility of collating and delivering the data for relevant families has resided with the appointed Taxonomic Specialists (see Table 1), while Associate Specialists deserve due credit for their important contributions at various levels, including particular geographic regions or (across) taxonomic groups (see Table 3).

Data management tasks were taken care of by the *Fauna Europaea* project bureau. During the project phase (until 2004) a network of principal partners took care of the diverse management tasks: Zoological Museum Amsterdam (general management & system development), Zoological Museum of Copenhagen (data collation), National Museum of Natural History in Paris (data validation) and Museum and Institute of Zoology in Warsaw (Newly Associated States [NAS] extension). From the formal termination of the project in 2004 to 2013, all tasks were taken over by the Zoological Museum Amsterdam.

### Study area description

The study area covers the western Palaearctic, including the European mainland, Great Britain, the Macaronesian islands, Cyprus, Faroe Islands, Iceland, Svalbard, Franz Josef Land and Novaya Zemlya, but excluding Turkey, the Caucasus, western Kazakhstan, the Arabian Peninsula and North Africa (see Fig. 3).

### Design description

*Standards*. Group Coordinators and taxonomic specialists have been delivering the (sub)species names according to strict standards. The names provided by *Fauna Europaea* are *scientific names*. The taxonomic scope includes issues like, (1) the definition of criteria used to identify the accepted species-group taxa, (2) the hierarchy (classification scheme) for the accommodation of all accepted (sub)species, (3) relevant synonyms, and (4) the correct nomenclature. The *Fauna Europaea* 'Guidelines for Group Coordinators and Taxonomic Specialists' (Suppl. material 1) include the standards, protocols, scope and geographical limits and provide the instructions for the more than 400 taxonomic specialists contributing to the project.

*Data management*. The data records could either be entered offline into a preformatted MS-Excel worksheet or directly into the *Fauna Europaea* transaction database using an online browser interface. Since 2013 the data servers are hosted at the Museum für Naturkunde in Berlin, and an updated data entry tool is under development.

*Data set*. The *Fauna Europaea* basic data set consists of: accepted (sub)species names (including authorship), synonyms (including authorship), taxonomic hierarchy / classification, misapplied names (including misspellings and alternative taxonomic views), homonym annotations, expert details, European distribution (at the level of country or major island), global distribution (only for European species), taxonomic reference (optional), occurrence reference (optional).

### Funding

*Fauna Europae* a was funded by the European Commission under the Fifth Framework Programme and contributed to the Support for Research Infrastructures work programme with Thematic Priority Biodiversity (EVR1-1999-20001) for a period of four years (1 March 2000 – 1 March 2004), including a short 'NAS extension', allowing EU candidate accession countries to participate. Follow-up support was given by the EC-FP5 EuroCAT project (EVR1-CT-2002-20011), by the EC-FP6 ENBI project (EVK2-CT-2002-20020), by the EC-FP6 EDIT project (GCE 018340), by the EC-FP7 PESI project (RI-223806) and by the EC-FP7 ViBRANT project (RI-261532). Continued management and hosting of the *Fauna Europaea* services was supported by the University of Amsterdam (Zoological Museum Amsterdam) and SARA/Vancis. Recently, the hosting of *Fauna Europaea* was taken over by the Museum für Naturkunde in Berlin, supported by the EC-FP7 EU BON project (grant agreement №308454).

Additional support for preparing the Diptera–Brachycera data set was received through the numerous institutions allowing for the proper allocation of time by the taxonomic specialists.

## Sampling methods

### Study extent

See relevant sections on coverage.

### Sampling description

*Fauna Europaea* data have been assembled by the principal taxonomic specialists based on their individual expertise, which includes studies of the literature, collection research, and field sampling. In total 476 taxonomic specialists contributed taxonomic and/or faunistic information for *Fauna Europaea*. The vast majority of the experts are from Europe (including EU non-member states). As a unique feature, *Fauna Europaea* funds were set aside for paying/compensating for the work of taxonomic specialists and Group Coordinators (around five Euro per species).

**Figure 1.**
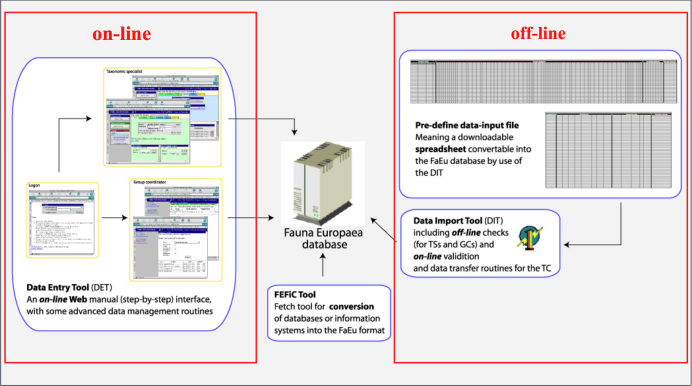
*Fauna Europaea* on-line (browser interfaces) and off-line (spreadsheets) data entry tools.

To facilitate data transfer and data import, sophisticated on-line (web interfaces) and off-line (spreadsheets) data-entry routines were built, well integrated within an underlying central *Fauna Europaea* transaction database (see Fig. 1). This included advanced batch data import routines and utilities to display and monitor the data processing within the system. In retrospect, it seems that the off-line submission of data was probably the best for bulk import during the project phase, while the on-line tool was preferred to enter modifications in later versions. This data management system worked well until its replacement in 2013.

A first release of *Fauna Europaea* via the web-portal was presented on 27 September 2004, whereas the most recent release (version 2.6.2) was launched on 29 August 2013. An overview of *Fauna Europaea* releases can be found at: http://www.faunaeur.org/about_fauna_versions.php.

### Quality control

*Fauna Europaea* data are unique in the sense that they are fully expert-based. Selecting leading experts for all groups provided a principal assurance of the systematic reliability and consistency of the *Fauna Europaea* data.

Further, all *Fauna Europaea* data sets have been intensively reviewed at regional and thematic validation meetings, at review sessions at taxonomic symposia (for some groups), by *Fauna Europaea* Focal Points (during the FaEu-NAS and PESI projects) and by various end-users sending annotations using the web form at the web-portal. Additional validation on gaps and correct spellings was effected by the validation office at the National Museum of Natural History in Paris.

Checks on technical and logical correctness of the data were implemented by the data entry tools, including around 50 'Taxonomic Integrity Rules'. This validation tool proved to be of considerable value for both the taxonomic specialists and project management, and significantly contributed to the preparation of a remarkably clean and consistent data set.

This thorough review procedure makes *Fauna Europaea* the most scrutinised data set in its domain. In general we expected to get taxonomic data for 99.3% of the known European fauna directly after the initial release of *Fauna Europaea* (de Jong et al. 2014). The faunistic coverage is not quite as good, but is nevertheless 90-95% of the total fauna. Currently, for the Diptera–Brachycera the taxonomic completeness is considered to be around 93% (see Table 1).

To optimise the use and implementation of a uniform and correct nomenclature, a cross-referencing of the *Fauna Europaea* Diptera data-set with relevant nomenclators, including Systema Dipterorum, is recommended, following the global efforts on establishing a so-called 'Global Names Architecture' (Pyle and Michel 2008).

### Step description

By evaluating team structure and procedures (data-entry, validation, updating, etc.), clear definitions of roles of users and user-groups in relation to the taxonomic classification were established, including ownership and read/write privileges. In addition, guidelines on common data exchange formats and codes have been issued (see also Suppl. material 1).

## Geographic coverage

### Description

**Figure 2.**
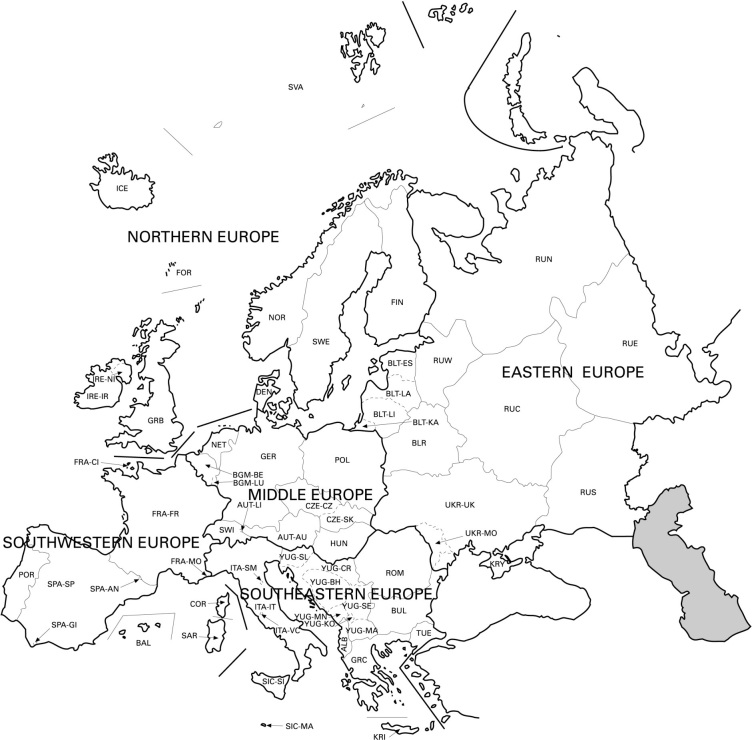
*Fauna Europaea* TDWG areas.

**Figure 3.**
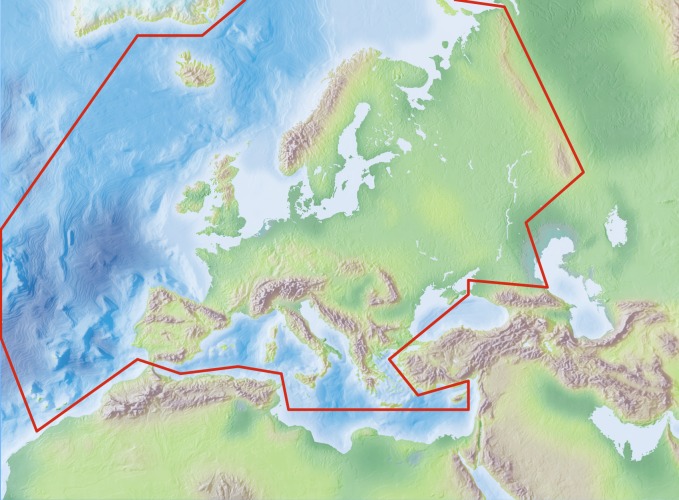
*Fauna Europaea* geographic coverage ('minimal Europe').

Species and subspecies distributions in *Fauna Europaea* are registered at least at the level of (political) country. For this purpose the FaEu geographical system basically follows the TDWG standards (see Fig. 2). The area studied covers the western Palaearctic, including the European mainland, Great Britain, the Macaronesian islands, Cyprus, Faroe Islands, Iceland, Svalbard, Franz Josef Land and Novaya Zemlya, but excluding Turkey, the Caucasus, western Kazakhstan, the Arabian Peninsula and North Africa (see Fig. 3).

The focus is on species (or subspecies) of European multicellular animals of land and freshwater environments. Species in brackish waters, occupying the marine/freshwater or marine/terrestrial transition zones, are generally excluded.

### Coordinates

Mediterranean and Arctic Islands Latitude; Atlantic Ocean (Mid-Atlantic Ridge) and Ural Longitude.

## Taxonomic coverage

### Description

The *Fauna Europaea* database contains the scientific names of all living European land and freshwater animal species, including numerous groups at various hierarchical levels, and the most important synonyms. More details about the conceptual background of *Fauna Europaea* and standards followed are described above.

**Figure 4.**
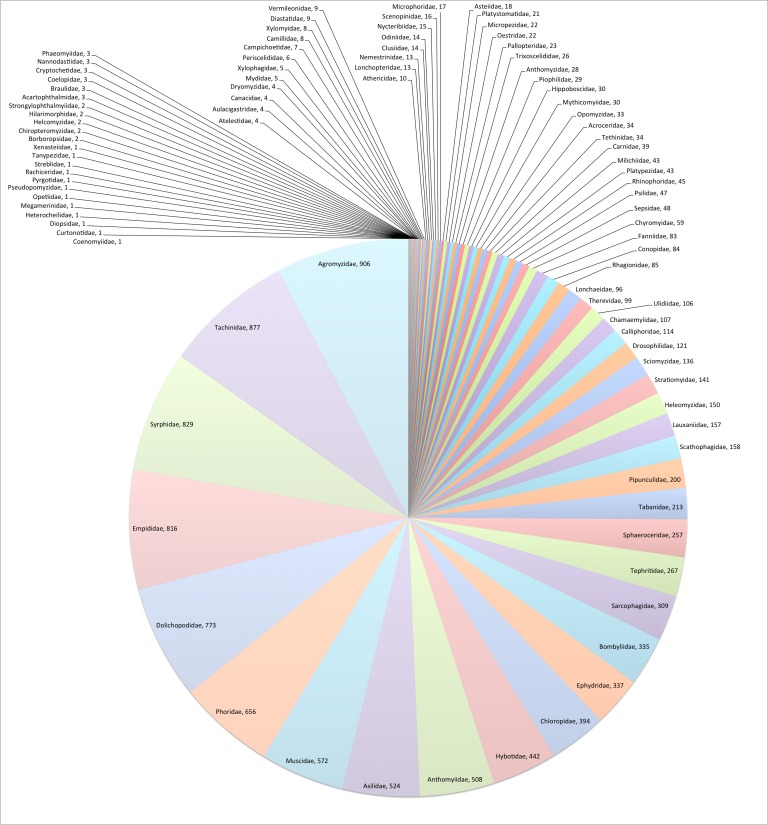
*Fauna Europaea* Diptera–Brachycera species per family. See Table 1 for family statistics. For full resolution see Suppl. material 2.

This data paper covers the Diptera–Brachycera content of *Fauna Europaea*, including 96 families, 11,751 species, 179 subspecies and 2,233 (sub)specific synonyms (see Fig. 4). Higher ranks are given below, the species list can be downloaded from the Fauna Europaea portal (see: Data resources).

Some recent changes in the classification of Diptera–Brachycera will be effectuated in the next version. This includes the merging of the families Canacidae and Tethinidae into a single family, Canacidae (the older family group name) (McAlpine 2007, Munari and Mathis 2010) and the splitting of the Calliphoridae (s.lat.) into Calliphoridae (s.str.) and Rhiniidae in accordance with what is now current practice (Pape et al. 2011, Pape and Evenhuis 2013).

### Taxa included

**Table:** 

Rank	Scientific Name	Common Name
kingdom	Animalia	animals
subkingdom	Eumetazoa	
phylum	Arthropoda	arthropods
subphylum	Hexapoda	hexapods
class	Insecta	insects
order	Diptera	true flies
suborder	Brachycera	
family	Acartophthalmidae	
family	Acroceridae	
family	Agromyzidae	
family	Anthomyiidae	
family	Anthomyzidae	
family	Asilidae	
family	Asteiidae	
family	Atelestidae	
family	Athericidae	
family	Aulacigastridae	
family	Bombyliidae	
family	Borboropsidae	
family	Braulidae	
family	Calliphoridae	
family	Camillidae	
family	Campichoetidae	
family	Canacidae	
family	Carnidae	
family	Chamaemyiidae	
family	Chiropteromyzidae	
family	Chloropidae	
family	Chyromyidae	
family	Clusiidae	
family	Cnemospathididae	
family	Coelopidae	
family	Coenomyidae	
family	Coenomyiidae	
family	Conopidae	
family	Cryptochetidae	
family	Curtonotidae	
family	Diastatidae	
family	Diopsidae	
family	Dolichopodidae	
family	Drosophilidae	
family	Dryomyzidae	
family	Empididae	
family	Ephydridae	
family	Fanniidae	
family	Gasterophilidae	
family	Helcomyzidae	
family	Heleomyzidae	
family	Heterocheilidae	
family	Hilarimorphidae	
family	Hippoboscidae	
family	Hybotidae	
family	Hypodermatidae	
family	Lauxaniidae	
family	Lonchaeidae	
family	Lonchopteridae	
family	Megamerinidae	
family	Micropezidae	
family	Microphoridae	
family	Milichiidae	
family	Muscidae	
family	Mydidae	
family	Mythicomyiidae	
family	Nannodastiidae	
family	Nemestrinidae	
family	Neottiophilidae	
family	Nycteribiidae	
family	Odiniidae	
family	Oestridae	
family	Opetiidae	
family	Opomyzidae	
family	Otitidae	
family	Pallopteridae	
family	Periscelididae	
family	Phaeomyiidae	
family	Phoridae	
family	Piophilidae	
family	Pipunculidae	
family	Platypezidae	
family	Platystomatidae	
family	Pseudopomyzidae	
family	Psilidae	
family	Pyrgotidae	
family	Rachiceridae	
family	Rhagionidae	
family	Rhinophoridae	
family	Sarcophagidae	
family	Scathophagidae	
family	Scenopinidae	
family	Sciomyzidae	
family	Sciomyzidae	
family	Sepsidae	
family	Solvidae	
family	Sphaeroceridae	
family	Stenomicridae	
family	Stratiomyidae	
family	Streblidae	
family	Strongylophthalmyiidae	
family	Syrphidae	
family	Tabanidae	
family	Tachinidae	
family	Tanypezidae	
family	Tephritidae	
family	Tethinidae	
family	Therevidae	
family	Thyreophoridae	
family	Trixoscelididae	
family	Ulidiidae	
family	Vermileonidae	
family	Xenasteiidae	
family	Xylomyidae	
family	Xylophagidae	
subfamily	Achanthipterinae	
subfamily	Agromyzinae	
subfamily	Anthomyzinae	
subfamily	Anthracinae	
subfamily	Antoniinae	
subfamily	Apocleinae	
subfamily	Asilinae	
subfamily	Azeliinae	
subfamily	Bombyliinae	
subfamily	Callomyiinae	
subfamily	Calobatinae	
subfamily	Canacinae	
subfamily	Chalarinae	
subfamily	Chamaemyiinae	
subfamily	Clusiinae	
subfamily	Clusiodinae	
subfamily	Coelopinae	
subfamily	Coenosiinae	
subfamily	Copromyzinae	
subfamily	Cremifaniinae	
subfamily	Cythereinae	
subfamily	Dacinae	
subfamily	Dasiopinae	
subfamily	Dasypogoninae	
subfamily	Dexiinae	
subfamily	Discomyzinae	
subfamily	Drosophilinae	
subfamily	Ecliminae	
subfamily	Ephydrinae	
subfamily	Exoristinae	
subfamily	Gasterophilinae	
subfamily	Gymnomyzinae	
subfamily	Heleomyzinae	
subfamily	Heteromyzinae	
subfamily	Hirmoneurinae	
subfamily	Hydrelliinae	
subfamily	Hypodermatinae	
subfamily	Ilytheinae	
subfamily	Laphriinae	
subfamily	Laphystiinae	
subfamily	Leptogastrinae	
subfamily	Leptomydinae	
subfamily	Limosininae	
subfamily	Lomatiinae	
subfamily	Lonchaeinae	
subfamily	Madizinae	
subfamily	Micropezinae	
subfamily	Microsaniinae	
subfamily	Milichiinae	
subfamily	Miltogramminae	
subfamily	Muscinae	
subfamily	Mydaeinae	
subfamily	Nemestrininae	
subfamily	Neottiophilinae	
subfamily	Nephrocerinae	
subfamily	Oestrinae	
subfamily	Oligodraninae	
subfamily	Orygmatinae	
subfamily	Otitinae	
subfamily	Paramacronychiinae	
subfamily	Periscelidinae	
subfamily	Phaoniinae	
subfamily	Phasiinae	
subfamily	Phthiriinae	
subfamily	Phycinae	
subfamily	Phytomyzinae	
subfamily	Piophilinae	
subfamily	Pipunculinae	
subfamily	Platypezinae	
subfamily	Platystomatinae	
subfamily	Sarcophaginae	
subfamily	Sepsinae	
subfamily	Sphaerocerinae	
subfamily	Steganinae	
subfamily	Stenomicrinae	
subfamily	Stenopogoninae	
subfamily	Stichopogoninae	
subfamily	Suilliinae	
subfamily	Syllegomydinae	
subfamily	Tachininae	
subfamily	Taeniapterinae	
subfamily	Tephritinae	
subfamily	Therevinae	
subfamily	Toxophorinae	
subfamily	Trichopsidiinae	
subfamily	Trypetinae	
subfamily	Ulidiinae	
subfamily	Usiinae	
tribe	Adramini	
tribe	Andrenosomini	
tribe	Anthracini	
tribe	Aphoebantini	
tribe	Apolysini	
tribe	Atherigonini	
tribe	Atissini	
tribe	Atomosiini	
tribe	Azeliini	
tribe	Bombyliini	
tribe	Borboropsini	
tribe	Canacini	
tribe	Carpomyini	
tribe	Cecidocharini	
tribe	Cephaliini	
tribe	Cephalopsini	
tribe	Ceratitidini	
tribe	Chamaemyiini	
tribe	Chiropteromyzini	
tribe	Coelopini	
tribe	Coenosiini	
tribe	Conophorini	
tribe	Cyrtopogonini	
tribe	Dacini	
tribe	Dagini	
tribe	Dasypogonini	
tribe	Dichaetomyiini	
tribe	Dioctriini	
tribe	Discocerinini	
tribe	Discomyzini	
tribe	Dithrycini	
tribe	Drosophilini	
tribe	Dryxini	
tribe	Dynomiellini	
tribe	Eginiini	
tribe	Ephydrini	
tribe	Euarestini	
tribe	Eudorylini	
tribe	Exoprosopini	
tribe	Gerontini	
tribe	Gitonini	
tribe	Glumini	
tribe	Gymnomyzini	
tribe	Hecamedini	
tribe	Heleomyzini	
tribe	Heteromyzini	
tribe	Hyadinini	
tribe	Hydrelliini	
tribe	Ilytheini	
tribe	Incertaesedistephritinini	
tribe	Isopogonini	
tribe	Laphriini	
tribe	Leucopini	
tribe	Limnophorini	
tribe	Lipochaetini	
tribe	Lipsanini	
tribe	Lomatiini	
tribe	Microcephalopsini	
tribe	Molobratiini	
tribe	Muscini	
tribe	Mycetaulini	
tribe	Myennidini	
tribe	Myopitini	
tribe	Nidomyiini	
tribe	Noeetini	
tribe	Notiphilini	
tribe	Ochtherini	
tribe	Oecotheini	
tribe	Orbelliini	
tribe	Otitini	
tribe	Parydrini	
tribe	Phaoniini	
tribe	Piophilini	
tribe	Pipunculini	
tribe	Plesiocerini	
tribe	Psilopini	
tribe	Reinwardtiini	
tribe	Scatellini	
tribe	Seiopterini	
tribe	Steganini	
tribe	Stenopogonini	
tribe	Stomoxyini	
tribe	Suilliini	
tribe	Tephrellini	
tribe	Tephritini	
tribe	Terelliini	
tribe	Tomosvaryellini	
tribe	Toxophorini	
tribe	Trixoscelidini	
tribe	Trypetini	
tribe	Typopsilopini	
tribe	Ulidiini	
tribe	Usiini	
tribe	Villini	
tribe	Xeramoebini	
tribe	Xyphosiini	
tribe	Zaceratini	
subtribe	Acletoxina	
subtribe	Carpomyina	
subtribe	Chetostomatina	
subtribe	Drosophilina	
subtribe	Gitonina	
subtribe	Leucophengina	
subtribe	Nitrariomyiina	
subtribe	Oedaspidina	
subtribe	Piophilina	
subtribe	Plioreoceptina	
subtribe	Steganina	
subtribe	Tephrellina	
subtribe	Thyreophorina	
subtribe	Trypetina	

## Temporal coverage

**Living time period:** Currently living multicellular, terrestrial and freshwater animals in stable populations, largely excluding (1) rare / irregular immigrants, (2) alien / invasive species, (3) accidental or deliberate releases of exotic (pet)species, (4) domesticated animals, (5) non-native species imported and released for bio-control or (6) non-native species largely confined to hothouses..

## Usage rights

### Use license

Open Data Commons Attribution License

### IP rights notes

*Fauna Europaea* data are licensed under CC BY SA version 4.0. The property rights of experts over their data is covered under the SMEBD conditions. For more copyrights and citation details see: http://www.faunaeur.org/copyright.php.

## Data resources

### Data package title

Fauna Europaea - Diptera-Brachycera

### Resource link

http://www.faunaeur.org/Data_papers/FaEu_Diptera-Brachycera_2.6.2.zip

### Alternative identifiers

http://www.faunaeur.org/experts.php?id=92

### Number of data sets

2

### Data set 1.

#### Data set name

Fauna Europaea - Diptera-Brachycera version 2.6.2 - species

#### Data format

CSV

#### Number of columns

25

#### Character set

UTF-8

#### Download URL

http://www.faunaeur.org/Data_papers/FaEu_Diptera-Brachycera_2.6.2.zip

#### Description

**Data set 1.** 

Column label	Column description
datasetName	The name identifying the data set from which the record was derived (http://rs.tdwg.org/dwc/terms/datasetName).
version	Release version of data set.
versionIssued	Issue data of data set version.
rights	Information about rights held in and over the resource (http://purl.org/dc/terms/rights).
rightsHolder	A person or organization owning or managing rights over the resource (http://purl.org/dc/terms/rightsHolder).
accessRights	Information about who can access the resource or an indication of its security status (http://purl.org/dc/terms/accessRights).
taxonID	An identifier for the set of taxon information (http://rs.tdwg.org/dwc/terms/taxonID)
parentNameUsageID	An identifier for the name usage of the direct parent taxon (in a classification) of the most specific element of the scientificName (http://rs.tdwg.org/dwc/terms/parentNameUsageID).
scientificName	The full scientific name, with authorship and date information if known (http://rs.tdwg.org/dwc/terms/scientificName).
acceptedNameUsage	The full name, with authorship and date information if known, of the currently valid (zoological) taxon (http://rs.tdwg.org/dwc/terms/acceptedNameUsage).
originalNameUsage	The original combination (genus and species group names), as firstly established under the rules of the associated nomenclaturalCode (http://rs.tdwg.org/dwc/terms/originalNameUsage).
family	The full scientific name of the family in which the taxon is classified (http://rs.tdwg.org/dwc/terms/family).
familyNameId	An identifier for the family name.
genus	The full scientific name of the genus in which the taxon is classified (http://rs.tdwg.org/dwc/terms/genus).
subgenus	The full scientific name of the subgenus in which the taxon is classified. Values include the genus to avoid homonym confusion (http://rs.tdwg.org/dwc/terms/subgenus).
specificEpithet	The name of the first or species epithet of the scientificName (http://rs.tdwg.org/dwc/terms/specificEpithet).
infraspecificEpithet	The name of the lowest or terminal infraspecific epithet of the scientificName, excluding any rank designation (http://rs.tdwg.org/dwc/terms/infraspecificEpithet).
taxonRank	The taxonomic rank of the most specific name in the scientificName (http://rs.tdwg.org/dwc/terms/infraspecificEpithet).
scientificNameAuthorship	The authorship information for the scientificName formatted according to the conventions of the applicable nomenclaturalCode (http://rs.tdwg.org/dwc/terms/scientificNameAuthorship).
authorName	Author name information
namePublishedInYear	The four-digit year in which the scientificName was published (http://rs.tdwg.org/dwc/terms/namePublishedInYear).
Brackets	Annotation if authorship should be put between parentheses.
nomenclaturalCode	The nomenclatural code under which the scientificName is constructed (http://rs.tdwg.org/dwc/terms/nomenclaturalCode).
taxonomicStatus	The status of the use of the scientificName as a label for a taxon (http://rs.tdwg.org/dwc/terms/taxonomicStatus).
resourceDescription	An account of the resource, including a data-paper DOI (http://purl.org/dc/terms/description)

### Data set 2.

#### Data set name

Fauna Europaea - Diptera-Brachycera version 2.6.2 - hierarchy

#### Data format

CSV

#### Number of columns

12

#### Character set

UTF-8

#### Download URL

http://www.faunaeur.org/Data_papers/FaEu_Diptera-Brachycera_2.6.2.zip

#### Description

**Data set 2.** 

Column label	Column description
datasetName	The name identifying the data set from which the record was derived (http://rs.tdwg.org/dwc/terms/datasetName).
version	Release version of data set.
versionIssued	Issue data of data set version.
rights	Information about rights held in and over the resource (http://purl.org/dc/terms/rights).
rightsHolder	A person or organization owning or managing rights over the resource (http://purl.org/dc/terms/rightsHolder).
accessRights	Information about who can access the resource or an indication of its security status (http://purl.org/dc/terms/accessRights).
taxonName	The full scientific name of the higher-level taxon
scientificNameAuthorship	The authorship information for the scientificName formatted according to the conventions of the applicable nomenclaturalCode (http://rs.tdwg.org/dwc/terms/scientificNameAuthorship).
taxonRank	The taxonomic rank of the most specific name in the scientificName (http://rs.tdwg.org/dwc/terms/infraspecificEpithet).
taxonID	An identifier for the set of taxon information (http://rs.tdwg.org/dwc/terms/taxonID)
parentNameUsageID	An identifier for the name usage of the direct parent taxon (in a classification) of the most specific element of the scientificName (http://rs.tdwg.org/dwc/terms/parentNameUsageID).
resourceDescription	An account of the resource, including a data-paper DOI (http://purl.org/dc/terms/description)

## Additional information

In the very last phase of the Diptera-Brachycera paper preparation, we received the sad news that one of our respected Fauna Europaea experts on Tachinidae and co-author of this paper, Vera Andreevna Richter, passed away at the age of 79 years. A short obituary can be found here: Suppl. material 3.

## Supplementary Material

Supplementary material 1 Fauna Europaea Guidelines for Group Coordinators and Taxonomic Specialists Data type: pdf
File: oo_3494.pdf Nicolas Bailly, Verner Michelsen, Yde de Jong

Supplementary material 2 FaEu Diptera-Brachycera stats Data type: png
Brief description: This is a high-resolution version of Figure 4.
File: oo_32490.png Yde de Jong

Supplementary material 3 Vera Andreevna Richter short obituary Data type: pdf
File: oo_38602.pdf Dr. B.A. Korotyaev; Dr. O.G. Ovtshinnikova; Dr. V.A. Krivokhatsky

## Figures and Tables

**Table 1.** Taxonomic specialists per family for Diptera–Brachycera and their responsibilities. Expert replacements to be implemented for coming versions are given in Table 2. The actual numbers of databased species are given per family. For most families is also given an indication of the actual number of known/described species (showing a potential information gap) plus an estimate of the total number of existing species (i.e., described/known plus undescribed/undiscovered) for Europe.

TAXONOMY		EUROPE		
FAMILY	SPECIALIST(S)	DATABASED SPECIES (Fauna Europaea)	TOTAL RECORDED SPECIES (information-gap)	TOTAL ESTIMATED SPECIES (knowledge-gap)
Acartophthalmidae	Andrey L. Ozerov	3	3	3-4
Acroceridae	Emilia P. Nartshuk	34	34	34
Agromyzidae	Michel Martinez	906	910	>1200
Anthomyiidae	Verner Michelsen	508	508	570
Anthomyzidae	Jindřich Roháček	28	32	35
Asilidae	Fritz Geller-Grimm	524	584	•
Asteiidae	Miguel Carles-Tolrá	18	21	24
Atelestidae	Milan Chvála	4	3	3
Athericidae	Rudolf Rozkosny	10	10	10
Aulacigastridae	Miguel Carles-Tolrá	4	4	4
Bombyliidae	David J. Greathead [deceased] — Neal Evenhuis [follow-up]	335	335	355
Borboropsidae	Andrzej J. Woznica	2	2	2
Braulidae	Miguel Carles-Tolrá	3	3	3
Calliphoridae	Knut Rognes	114	115	130
Camillidae	Miguel Carles-Tolrá	8	9	10
Campichoetidae	Peter J. Chandler	7	7	7
Canacidae	Wayne N. Mathis	4	5	8
Carnidae	Andrej L. Ozerov	39	46	55
Chamaemyiidae	Stephen D. Gaimari	107	109	128
Chiropteromyzidae	Andrzej J. Woznica	2	2	2
Chloropidae	Emilia P. Nartshuk	394	412	475
Chyromyidae	Martin John Ebejer	59	64	74
Clusiidae	Jindřich Roháček & Bernhard Merz	14	14	15
Cnemospathididae	Andrzej J. Woźnica	0	1	1
Coelopidae	Rudolf Meier	3	3	3
Coenomyiidae	Rudolf Rozkosny	1	1	1
Conopidae	David K. Clements	84	84	90
Cryptochetidae	Emilia P. Nartshuk	3	3	3
Curtonotidae	Miguel Carles-Tolrá	1	1	1
Diastatidae	Peter J. Chandler	9	9	9
Diopsidae	Rudolf Meier	1	1	1
Dolichopodidae	Marc Pollet	773	796	900
Drosophilidae	Gerhard Bächli	121	122	•
Dryomyzidae	Miguel Carles-Tolrá	4	4	4
Empididae	Milan Chvála	816	860	•
Ephydridae	Tadeusz Zatwarnicki	337	340	340
Fanniidae	Adrian C. Pont	83	•	•
Helcomyzidae	Rudolf Meier	2	2	2
Heleomyzidae	Andrzej J. Woznica	150	152	165
Heterocheilidae	Rudolf Meier	1	1	1
Hilarimorphidae	Thomas Pape — Christian Kehlmaier [follow-up]	2	2	2
Hippoboscidae	Frederik T. Petersen	30	30	•
Hybotidae	Milan Chvála	442	470	•
Lauxaniidae	Bernhard Merz	157	•	•
Lonchaeidae	Miguel Carles-Tolrá — Thomas Pape [follow-up]	96	102	110
Lonchopteridae	Miroslav Barták	13	11	11
Megamerinidae	Andrej L. Ozerov	1	1	1
Micropezidae	Andrej L. Ozerov	22	22	25
Microphoridae	Milan Chvála	17	20	•
Milichiidae	Miguel Carles-Tolrá	43	45	48
Muscidae	Adrian C. Pont	572	•	•
Mydidae	David J. Greathead [deceased] — Torsten Dikow [follow-up]	5	6	8
Mythicomyiidae	David J. Greathead [deceased] — Neal Evenhuis [follow-up]	30	32	70
Nannodastiidae	Martin John Ebejer	3	3	4
Nemestrinidae	David J. Greathead [deceased] — Torsten Dikow [follow-up]	13	13	15
Nycteribiidae	Karel Hůrka [deceased] — Mihály Földvári [follow-up]	15	16	20
Odiniidae	Miguel Carles-Tolrá	14	15	16
Oestridae	Thomas Pape	22	22	22
Opetiidae	Peter J. Chandler	1	1	1
Opomyzidae	Jan-Willem van Zuijlen	33	33	36
Pallopteridae	Bernhard Merz — Miguel Carles-Tolrá [follow-up]	23	23	24
Periscelididae (incl. Stenomicridae)	Miguel Carles-Tolrá	6	7	9
Phaeomyiidae	Rudolf Rozkosny	3	3	3
Phoridae	Gisela Weber & Sabine Prescher	656	•	>1500
Piophilidae	Andrey L. Ozerov	29	31	35
Pipunculidae	Marc de Meyer — Christian Kehlmaier [follow-up]	200	205	230
Platypezidae	Peter J. Chandler	43	45	53
Platystomatidae	Valery A. Korneyev	21	21	25
Pseudopomyzidae	Bernhard Merz — Miguel Carles-Tolrá [follow-up]	1	1	1
Psilidae	Thomas Pape	47	49	55
Pyrgotidae	Bernhard Merz — Valery A. Korneyev [follow-up]	1	2	2
Rachiceridae	Rudolf Rozkosny	1	1	1
Rhagionidae	József Majer	85	85	90
Rhinophoridae	Thomas Pape	45	48	55
Sarcophagidae	Thomas Pape	309	•	350
Scathophagidae	Herman de Jong	158	•	•
Scenopinidae	Miguel Carles-Tolrá	16	16	18
Sciomyzidae	Rudolf Rozkosny	136	138	145
Sepsidae	Rudolf Meier	48	45	50
Sphaeroceridae	Jindřich Roháček	257	260	>270
Stratiomyidae	Rudolf Rozkosny	141	140	145
Streblidae	Karel Hůrka [deceased] — Mihály Földvári [follow-up]	1	1	2
Strongylophthalmyiidae	Thomas Pape	2	2	2
Syrphidae	Martin C. D. Speight	829	875	950
Tabanidae	Milan Chvála	213	220	•
Tachinidae	Hans-Peter Tschorsnig	877	897	920
Tanypezidae	Jindřich Roháček	1	1	1
Tephritidae	Valery A. Korneyev	267	267	275
Tethinidae	Lorenzo Munari	34	33	34
Therevidae	Kevin C. Holston	99	102	120
Trixoscelididae	Andrzej J. Woznica	26	27	40
Ulidiidae	Elena P. Kameneva & Lita Greve-Jensen	106	106	114
Vermileonidae	Thomas Pape — Christian Kehlmaier [follow-up]	9	9	25
Xenasteiidae	Miguel Carles-Tolrá	1	2	3
Xylomyidae	Rudolf Rozkosny	8	8	8
Xylophagidae	Rudolf Rozkosny	5	5	5

**Table 2.** Changes in group coordinatorship and taxonomic specialists for Diptera–Brachycera, which will take effect from Version 3.

FAMILY NAME	EXPERTS VERSIONS 1 & 2 (current)	Comment	EXPERTS VERSION 3 (future)	Comment
Bombyliidae	David J. Greathead	Deceased	Neal L. Evenhuis	
Mydidae	David J. Greathead	Deceased	Torsten Dikow	
Mythicomyiidae	David J. Greathead	Deceased	Neal L. Evenhuis	
Nemestrinidae	David J. Greathead	Deceased	Torsten Dikow	
Nycteribiidae	Karel Hůrka (Farkač 2005)	Deceased	Mihály Földvári	
Streblidae	Karel Hůrka (Farkač 2005)	Deceased	Mihály Földvári	
Hippoboscidae	Frederik T. Petersen	Resigned	Thomas Pape	
Lonchaeidae	Miguel Carles-Tolrá	Resigned	Iain MacGowan	
Lauxaniidae	Bernhard Merz	Resigned	Stephen D. Gaimari	
Pallopteridae	Bernhard Merz	Resigned	Miguel Carles-Tolrá	
Pseudopomyzidae	Bernhard Merz	Resigned	Miguel Carles-Tolrá	
Pyrgotidae	Bernhard Merz	Resigned	Valery A. Korneyev	
Hilarimorphidae	Thomas Pape	Resigned	Christian Kehlmaier	
Vermileonidae	Thomas Pape	Resigned	Christian Kehlmaier	
Pipunculidae	Marc de Meyer	Resigned	Christian Kehlmaier	
Clusiidae	Jindřich Roháček & Bernhard Merz	BM Resigned	Jindřich Roháček	
Ulidiidae	Elena P. Kameneva & Lita Greve-Jensen	LGJ Resigned	Elena P. Kameneva	
	Paul Beuk (version 2)	Resigned	Thomas Pape	Group Coordinatorship

**Table 3.** Associated Specialists for Diptera–Brachycera and their responsibilities.

TAXONOMIC GROUP or GEOGRAPHIC AREA	SPECIALIST
<Norway>	Lita Greve-Jensen
<Spain>, <Portugal>, <Andorra>	Miguel Carles-Tolrá
Lauxaniidae	Anatole I. Shatalkin
Bombyliidae, Mythicomyiidae	Neal L. Evenhuis
Tachinidae	Christer Bergström
Tachinidae	Pierfilippo Cerretti
Tachinidae	Vera Richter [deceased]
Tachinidae	Zdravko Hubenov
Tachinidae	Theo Zeegers
Tachinidae	Chris Raper
Tachinidae	Cezary Bystrowski
Tachinidae	Joachim Ziegler
Tachinidae	Jaromír Vaňhara
Tachinidae	Guy Van de Weyer
Chamaemyiidae	Vitali N. Tanasijtshuk

## References

[B863289] Byrd J. H., Castner J. L. (2000). Forensic Entomology: the utility of arthropods in legal investigations.

[B447149] Carles-Tolrá M., Cañete Saiz F. J. (2012). Primera cita de *Thyreophora
cynophila* (Panzer) para la provincia de Cuenca (España) (Diptera: Piophilidae: Thyreophorina). Boletín de la Sociedad Entomologica Aragonesa.

[B447139] Carles-Tolrá M., Rodríguez P. C., Verdú J. (2010). *Thyreophora
cynophila* (Panzer, 1794): collected in Spain 160 years after it was thought to be extinct (Diptera: Piophilidae: Thyreophorini). Boletín de la Sociedad Entomológica Aragonesa (S.E.A.).

[B826950] Catts E. P., Goff M. L. (1992). Forensic entomology in criminal investigations. Annual review of Entomology.

[B447109] Cole J. (1996). A second British site for *Prosopantrum
flavifrons* (Tonnoir & Malloch) (Diptera, Heleomyzidae). Entomologist's Monthly Magazine.

[B860550] Courtney G. W., Pape T., Skevington J. H., Sinclair B. J., Adler P., Foottit R. G. (2009). Biodiversity of Diptera. Insect Biodiversity: Science and Society.

[B861262] de Jong Yde, Verbeek Melina, Michelsen Verner, Bjørn Per de Place, Los Wouter, Steeman Fedor, Bailly Nicolas, Basire Claire, Chylarecki Przemek, Stloukal Eduard, Hagedorn Gregor, Wetzel Florian, Glöckler Falko, Kroupa Alexander, Korb Günther, Hoffmann Anke, Häuser Christoph, Kohlbecker Andreas, Müller Andreas, Güntsch Anton, Stoev Pavel, Penev Lyubomir (2014). Fauna Europaea – all European animal species on the web. BDJ.

[B863298] Disney R. H. L. (1994). Scuttle Flies: The Phoridae.

[B1235621] Farkač Jan (2005). In memoriam Prof. RNDr. Karel Hůrka, DrSc. (borne June 2, 1931, died May 25, 2004). Klapalekiana.

[B882885] Fontaine Benoît, van Achterberg Kees, Alonso-Zarazaga Miguel Angel, Araujo Rafael, Asche Manfred, Aspöck Horst, Aspöck Ulrike, Audisio Paolo, Aukema Berend, Bailly Nicolas, Balsamo Maria, Bank Ruud A., Belfiore Carlo, Bogdanowicz Wieslaw, Boxshall Geoffrey, Burckhardt Daniel, Chylarecki Przemysław, Deharveng Louis, Dubois Alain, Enghoff Henrik, Fochetti Romolo, Fontaine Colin, Gargominy Olivier, Lopez Maria Soledad Gomez, Goujet Daniel, Harvey Mark S., Heller Klaus-Gerhard, van Helsdingen Peter, Hoch Hannelore, De Jong Yde, Karsholt Ole, Los Wouter, Magowski Wojciech, Massard Jos A., McInnes Sandra J., Mendes Luis F., Mey Eberhard, Michelsen Verner, Minelli Alessandro, Nafrıa Juan M. Nieto, van Nieukerken Erik J., Pape Thomas, De Prins Willy, Ramos Marian, Ricci Claudia, Roselaar Cees, Rota Emilia, Segers Hendrik, Timm Tarmo, van Tol Jan, Bouchet Philippe (2012). New Species in the Old World: Europe as a Frontier in Biodiversity Exploration, a Test Bed for 21st Century Taxonomy. PLoS ONE.

[B1203776] Fontaine B, Bouchet P, van Achterberg K, Alonso-Zarazaga M, Araujo R, Asche M, Aspöck U, Audisio P, Aukema B, Bailly N, Balsamo M, Bank R A, Barnard P, Belfiore C, Bogdanowicz W, Bongers T, Boxshall G, Burckhardt D, Camicas J L, Chylarecki P, Crucitti P, Deharveng L, Dubois A, Enghoff H, Faubel A, Fochetti R, Gargominy O, Gibson O, Gibson R, Gómez López M S, Goujet D, Harvey M S, Heller K G, van Helsdingen P, Hoch H, de Jong H., de Jong Y, Karsholt O, Los W, Lundqvist L, Magowski W, Manconi R, Martens J, Massard J A, Massard-Geimer G, Mcinnes S J, Mendes L F, Mey E, Michelsen V, Minelli A, Nielsen C, Nieto Nafría J M, Nieukerken E J van, Noyes J, Pape T, Pohl H, De Prins W, Ramos M, Ricci C, Roselaar C, Rota E, Schmidt-Rhaesa A, Segers H, Zur Strassen R, Szeptycki A, Thibaud J M, Thomas A, Timm T, van Tol J, Vervoort W, Willmann R (2007). The European union’s 2010 target: Putting rare species in focus. Biological Conservation.

[B883860] Hoffmann Anke, Penner Johannes, Vohland Katrin, Cramer Wolfgang, Doubleday Robert, Henle Klaus, Kõljalg Urmas, Kühn Ingolf, Kunin William, Negro Juan José, Penev Lyubomir, Rodríguez Carlos, Saarenmaa Hannu, Schmeller Dirk, Stoev Pavel, Sutherland William, Tuama Éamonn Ó, Wetzel Florian, Häuser Christoph L. (2014). The need for an integrated biodiversity policy support process – Building the European contribution to a global Biodiversity Observation Network (EU BON). Nature Conservation.

[B860608] Hövemeyer K., Papp L, Darvas B (2000). Ecology of Diptera. Contributions to a Manual of Palaearctic Diptera (with special reference to flies of economie importance).

[B826940] Howard J. (2001). Nuisance flies around a landfill: Patterns of abundance and distribution. Waste Management and Research.

[B884213] Hulcr J., Pollet M., Ubik K., Vrkoč J. (2005). Exploitation of kairomones and synomones by Medetera spp. (Diptera: Dolichopodidae), predators of spruce bark beetles. European Journal of Entomology.

[B447119] Ismay J., Smith D. (1994). *Prosopantrum
flavifrons* (Tonnoir and Malloch) (Diptera, Heleomyzidae) new to Britain and the Northern Hemisphere. Dipterists Digest.

[B860647] Ismay J. W., Nartshuk E. P., Papp L., Darvas B. (2000). Family Chloropidae. Contributions to a manual of Palaearctic Diptera (with special reference to flies of economic importance).

[B880341] Koenig D. P., Young C. W. (2007). First observation of parasitic relations between big-headed flies, *Nephrocerus* zetterstedt (Diptera: Pipunculidae) and crane flies, *Tipula* Linnaeus (Diptera: Tipulidae: Tipulinae), with larval and puparial descriptions for the genus *Nephrocerus*. Proceedings of the Entomological Society of Washington.

[B826902] Krafsur E. S., Moon R. D. (1997). Bionomics of the face fly, *Musca
autumnalis*. Annual Review of Entomology.

[B807811] Lambkin C. L., Sinclair B. J., Pape T., Courtney G. W., Skevington J. H., Meier R., Yeates D. K., Blagoderov V., Wiegmann B. M. (2013). The phylogenetic relationships among infraorders and superfamilies of Diptera based on morphological evidence. Systematic Entomology.

[B447159] Martín-Vega D., Báez A., Michelsen V. (2010). Back from the dead: *Thyreophora
cynophila* (Panzer, 1798) (Diptera: Piophilidae) ‘globally extinct’ fugitive in Spain. Systematic Entomology.

[B438297] Mathis W. N., Zatwarnicki T. (1995). World catalog of shore flies (Diptera: Ephydridae). Memoirs of Entomology International.

[B862582] McAlpine David K. (2007). The surge flies (Diptera: Canacidae: Zaleinae) of Australasia and notes on tethinid-canacid morphology and relationships. Records of the Australian Museum.

[B863220] McLean I. F. G., Papp L., Darvas B. (2000). Beneficial Diptera and their role in decomposition. Contributions to a Manual of Palaearctic Diptera (with special reference to flies of economic importance).

[B862572] Munari L, Mathis W. N. (2010). World Catalog of the Family Canacidae (including Tethinidae) (Diptera), with keys to the supraspecific taxa. Zootaxa.

[B826892] Nartshuk E. P. (2000). Periodicity of outbreaks of the predatory fly *Thaumatomyia
notata* Mg. (Diptera, Chloropidae) and its possible reasons. Entomologicheskoe obozrenie.

[B882966] Pape T., Pape T., Bickel D., Meier R. (2009). Palaearctic Diptera — from Tundra to Desert. Diptera Diversity: Status, Challenges and Tools.

[B880143] Pape T., Evenhuis N. L. (2013). Notes on our family classification. http://www.diptera.org/ClassificationNotes.php.

[B882852] Pape T., Bickel D., Meier R. (2009). *Diptera Diversity: Status, Challenges and Tools*.

[B881246] Pape T., Blagoderov V., Mostovski M. B. (2011). Order DIPTERA Linnaeus, 1758. *In*: Zhang, Z.-Q. (Ed.) Animal biodiversity: An outline of higher-level classification and survey of taxonomic richness.. Zootaxa.

[B447169] Preisler J., Roháček J. (2012). New faunistic records of Heleomyzidae (Diptera) from the Czech Republic and Slovakia, and notes on the distribution of three rare *Suillia* species. Časopis Slezského zemského muzea (A).

[B1197448] Pyle RL, Michel E (2008). ZooBank: Developing a nomenclatural tool for unifying 250 years of biological information.. Zootaxa.

[B863307] Rivers D. B., Dahlem G. A. (2014). The Science of Forensic Entomology.

[B826882] Séguy E. (1950). La biologie des diptères. Encyclopédie entomologique, Serie A.

[B826872] Sherman R. A. (2001). Maggot therapy for foot and leg wounds. International Journal of Lower Extremity Wounds.

[B826862] Sherman R. A. (2002). Maggot vs conservative debridement therapy for the treatment of pressure ulcers. Wound Repair and Regeneration.

[B826838] Sherman R. A. (2003). Cohort study of maggot therapy for treating diabetic foot ulcers. Diabetes Care.

[B447179] Soszyńska-Maj A., Woźnica A. J. (2012). Uwagi na temat biologii, systematyki i rozmieszczenia Scoliocentra (Leriola) nigrinervis (Wahlgren, 1918) w Polsce i Europie (Diptera: Heleomyzidae). Dipteron.

[B826827] Stuke J. - H., Merz B. (2005). *Prosopantrum
flavifrons* (Tonnoir & Malloch, 1927) in Mitteleuropa nachgewiesen (Diptera: Heleomyzoidea s. l., Cnemospathidae). Studia dipterologica.

[B860574] Thompson F. C., Rotheray G., Papp L., Darvas B. (1998). Family Syrphidae. Contributions to a manual of Palaearctic Diptera (with special reference to flies of economic importance).

[B807775] Wiegmann B. M., Trautwein M. D., Winkler I. S., Barr N. B., Kin J. W., Lambkin C., Bertone M. A., Cassel B K., Bayless K. M., Heimberg A. M., Wheeler B. M., Peterson K. J., Pape T., Sinclair B. J., Skevington J. H., Blagoderov V., Caravas J., Kutty S. N., Schmidt-Ott U., Kampmeier G. E., Thompson F. C., Grimaldi D. A., Beckenbach A. T., Courtney G. W., Friedrich M., Meier R., Yeates D. K. (2011). Episodic radiations in the fly tree of life. Proceedings of the National Academy of Sciences.

[B826817] Yeates D. K., Wiegmann B. M. (1999). Congruence and controversy: toward a higher-level classification of Diptera. Annual Review of Entomology.

[B447191] Zaldivar Ezquerro C., Rodrìguez P. C., Gòmez Vargas J. (2011). *Thyreophora
cynophila* (Panzer, 1798) (Diptera: Piophilidae: Thyreophorini): distribution area in La Rioja (Spain). Boletín de la Sociedad Entomologica Aragonesa.

