# Supplementary figures and images for: Fauna Europaea: Diptera – Brachycera
Source: Biodivers Data J. 2015 Feb 20;(3):e4187. doi: 10.3897/BDJ.3.e4187 (PMC4339814; doi:10.3897/BDJ.3.e4187)

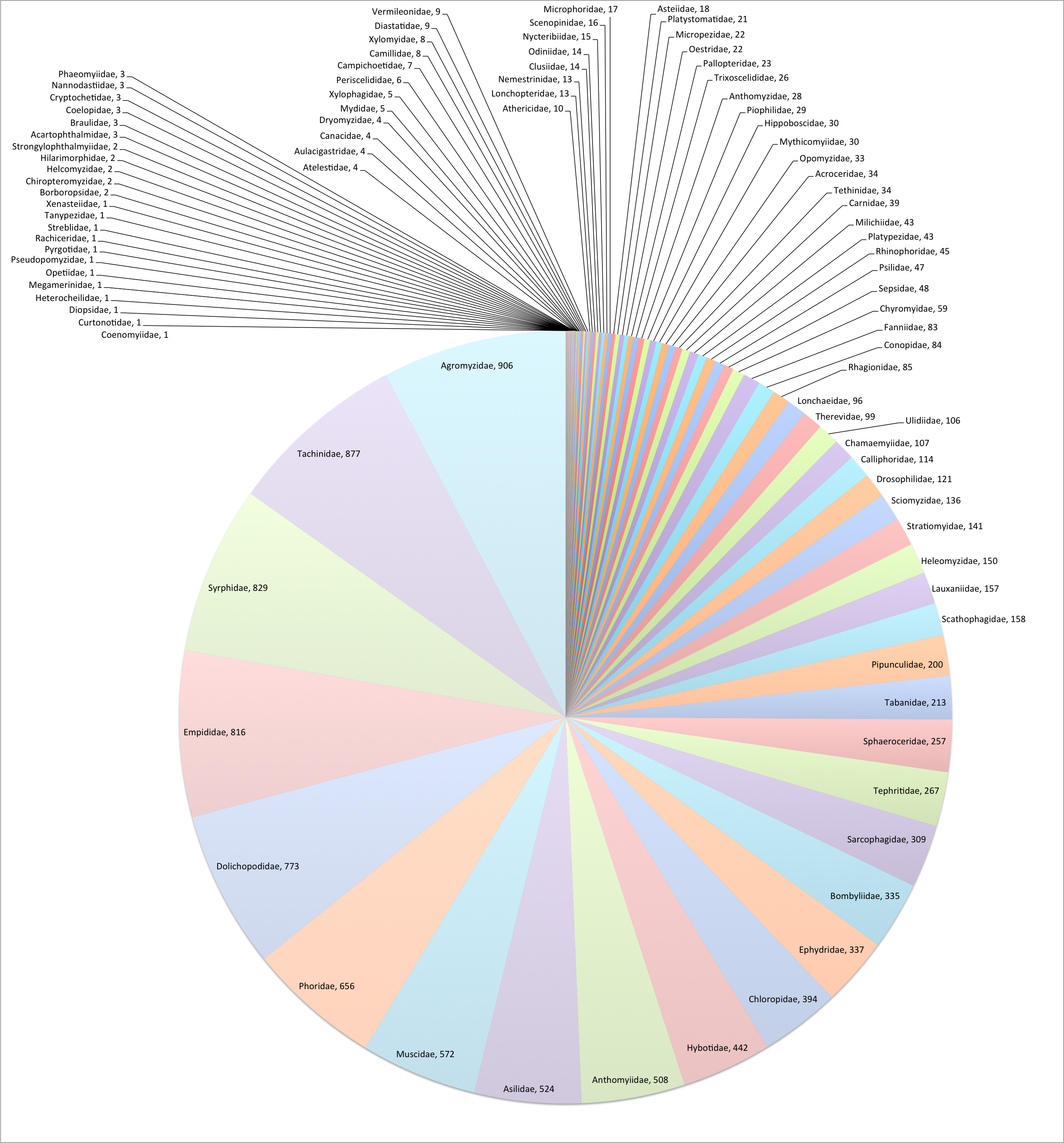

Supplement: Supplementary material 2 — FaEu Diptera-Brachycera stats [file biodiversity_data_journal-3-e4187-s002.png]
