# Supplementary material for: Fauna Europaea: Diptera – Brachycera
Source: Biodivers Data J. 2015 Feb 20;(3):e4187. doi: 10.3897/BDJ.3.e4187 (PMC4339814; doi:10.3897/BDJ.3.e4187)
Supplement: Supplementary material 3 — Vera Andreevna Richter short obituary [file biodiversity_data_journal-3-e4187-s003.pdf]

IN MEMORIAM

† Vera Andreevna RICHTER

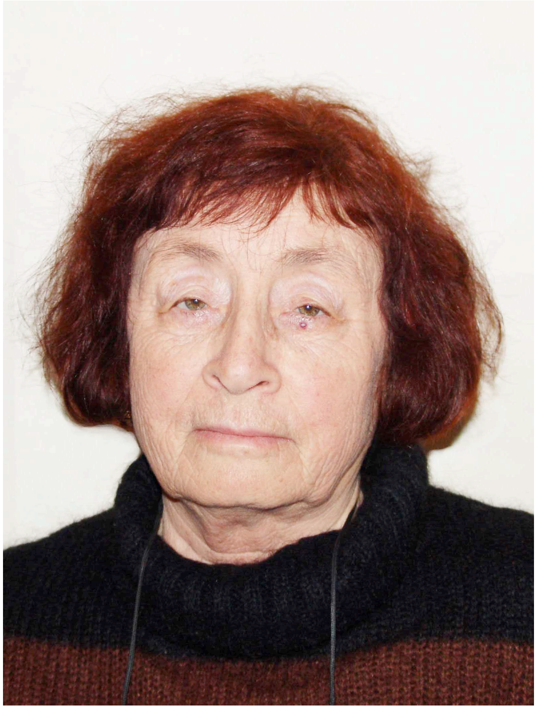

28 August 1936 – 07 February 2015

On February 7, 2015, Professor **Vera Andreevna RICHTER,** DSc in biology, a leading researcher in the Laboratory of Insect Systematics, Zoological Institute, Russian Academy of Sciences, St. Petersburg, Russia, died after a stroke at the age of 79 years. She was a prominent dipterist, a member of the Council and Presidium of the Russian Entomological Society, and Deputy Editor-in-Chief of the journal "*Entomologicheskoe Obozrenie*."

Her parents were prominent Soviet entomologists, Andrei Andreevich Richter, a specialist on Buprestidae, and Margarita Yervandovna Ter-Minassian, the author of the volumes of the "*Fauna of the USSR*" on Bruchidae and Rhynchitidae plus Attelabidae, and also of the books on the weevil subfamily Lixinae (formerly Cleoninae). V. A. RICHTER started her scientific activities as a student of the Entomology Department of the Leningrad State University, from which she graduated in 1958. By that time, she had published two papers on checkered beetles (family Cleridae). In 1958 she got a position as technician at the Zoological Institute in Leningrad and worked for five years with A. A. Stackelberg, doing her technical work and conducting studies of two families of Diptera, Asilidae and Tephritidae. In 1965 she presented her PhD dissertation, and in 1988 a dissertation on Tachinidae for the DSc in Biology degree. In 2003, she got a professorship.

Her main interests since 1960 included morphology, systematics, evolution and zoogeography of Palaearctic tachinids. Vera Andreevna has investigated a voluminous material of this group from the Caucasus, Middle Asia, Transbaikalia, Yakutia, Mongolia, Sakhalin and the Kurile Islands. She collected a lot of flies and beetles herself, and she has made an important contribution to the collection of the Zoological Institute.

Vera Andreevna RICHTER is the author of more than 240 publications, including a monograph of the robber-flies of the Caucasus, a chapter in the 'Manual of Palaearctic Diptera', and a large chapter in the 'Key to Insects of the Russian Far East'. Vera Andreevna has supervised 6 PhD dissertations on three fly families. She was a very knowledgeable biologist with profound love and deep sympathy for all living beings, and a high level of responsibility. She was a kind-hearted, very optimistic, easy to fake and set laughing lady, who was happy to look at flies until her last days. We will sorely miss her.

Dr. B.A. Korotyaev (baris@zin.ru) & Dr. O.G. Ovtshinnikova (brach@zin.ru)

Photo by Dr. V.A. Krivokhatsky

Zoological Institute, Russian Academy of Science, St. Petersburg 199034, Russia
